# Sleep, Mood, and Nutrition Patterns of Postmenopausal Women Diagnosed with Major Depressive Disorder by Menopause Periods

**DOI:** 10.3390/life14060775

**Published:** 2024-06-19

**Authors:** Cansu Kabadayı Demir, Sinem Bayram, Beril Köse, Esra Köseler Beyaz, Esen Yeşil

**Affiliations:** Nutrition and Dietetics Department, Faculty of Health Sciences, Baskent University, Ankara 06790, Türkiye; dytcansukabadayi@gmail.com (C.K.D.); berilyilmaz@baskent.edu.tr (B.K.); esezer@baskent.edu.tr (E.Y.)

**Keywords:** sleep, mood, nutrition, menopause, women

## Abstract

Menopausal women experience changes in ovarian activity and fluctuating hormone levels. The aim of this study was to detect ongoing sleep and nutritional problems in postmenopausal women. This study was conducted with 62 postmenopausal women who had been diagnosed with major depressive disorder, were aged 42–64, were not dieting for at least 1 month, and had been using antidepressants for at least 6 months. The Pittsburgh Sleep Quality Index and Depression Anxiety Stress Scale–42 were used. Anthropometric measurements were taken and body composition analysis was performed. The prevalence of obesity and overweight were 12.9% and 71%, respectively. Hot flashes, night sweats, and mood swing were more common in those who had been through menopause for <5 years. Also, the PSQI, Depression, and Stress scores of those women were higher. Women who had been menopausal for ≥5 years had a higher BMI, waist/hip ratio, and fat mass and better sleep quality and lower daytime dysfunction according to the PSQI. Energy and fat intake were higher, while protein, vitamin C, and calcium intakes were lower in women who had been menopausal for ≥5 years. It is crucial for healthcare professionals to review approaches for early and late menopausal periods and to individualize treatment options, especially in patients whose symptoms persist.

## 1. Introduction

Amenorrhea (absence of menstruation) observed for 12 months without any pathological cause is a sign of completion of the menstrual cycle [1]. Postmenopausal women experience a physiological deterioration in hypothalamic–pituitary–ovarian axis function associated with a progressive change in ovarian activity and fluctuating hormone levels, which can lead to sleep, mood, and nutritional disorders [2]. Sex steroids are able to modify several functions, including behavior, sleep, mood, pain, and coordination, amongst others [3]. Estrogen hormone plays a role in the regulation of serotonin, dopamine, and norepinephrine receptors, which are effective neurotransmitters in depression. Estrogen acts similarly to antidepressant treatment—in other words the opposite of stress—and prevents the formation of free radicals and proinflammatory markers that trigger depression by increasing mitochondrial activity [4]. 

Women are at a higher risk of developing depression, stress, anxiety, and emotional distress during menopausal transition. Women’s menstrual cycles, postpartum period, and transition to menopause increase the tendency to depression. Cardiovascular problems, vasomotor symptoms, sleep problems, and a stressful life are some of the points associated with depression during menopause [5].

Sleep disorders, night awakenings, hypersomnia, snoring, and apnea, which is one of the most important health problems of postmenopausal periods, can be observed in 40–60% of women. Especially if there are accompanying vasomotor symptoms (symptoms of night sweats and hot flashes), sleep problems are frequently observed [6]. These depressed mood and sleep problems (insomnia, night-time awakening, or waking early) are associated with some eating disorders [7].

Maintaining a balanced and varied diet is one of the main factors for a healthy lifestyle. It is vital to provide adequate energy and nutritional resources for both women’s health for generations and to reduce the risk of many chronic diseases in advancing age [8]. Although dietary food intake and sleep quality has been questioned so far, the relationship between these parameters and depression according to the mean time elapsed since menopause has rarely been emphasized. The aim of this study is to detect ongoing sleep and nutritional problems in postmenopausal women, despite antidepressant use. It has been hypothesized that as women’s menopause period increases, sleep and nutrition problems increase.

## 2. Materials and Methods

### 2.1. Participants 

This study was conducted with a total of 65 women, but since we had problems with 3 women continuing in the study, they were not included in order not to affect the results. The study was conducted with postmenopausal women diagnosed with major depressive disorder, aged 42–64, who applied to a nutrition counseling center between February and October 2021. The participants had not been dieting for at least 1 month, had menopausal status, and had been using antidepressants for at least 6 months. Individuals who had received hormone replacement therapy, had gynecological diseases, or diseases that could affect sleep quality were not included. The sample size for the study was determined by G*Power analysis. Based on percentage measurement values related to the methods studied in the literature, a 0.8 effect size, 80% power, and 0.05 error margin were used with G*Power to determine a total sample size of at least 60 women. For this study, “Ethics Committee Approval” from the Baskent University Ethics Committee’s decision dated 8 January 2020 and numbered 20/07 and a written consent form stating that they volunteered at the beginning of the study were obtained from each individual. 

### 2.2. Study design and Questionnaires

The women were questioned on how long it had been since their last menstrual cycle. Menopause was defined as the absence of menses for 12 consecutive months. Each individual included in this study was first given information about the study. Individuals who agreed to participate in the study read and signed the “Volunteer Consent Form”, which was approved by the ethics committee at our institution.

In the questionnaire form, 40 questions were asked to determine the demographic and menopausal characteristics of individuals. The questionnaire form was recorded by “face-to-face interview method”. In order to determine the daily energy and nutrient consumption of individuals, food records were taken for 3 days. 

The Pittsburgh Sleep Quality Index (PSQI) is a self-rated questionnaire which assesses sleep quality and disturbances over a 1-month time interval. In this study, the Turkish version of the PSQI, which was adapted by Agargun et al. [9], was used, and a Cronbach’s alpha value of the scale of 0.79 was found (Cronbach’s alpha = 0.79). 

In order to determine the individual and social effects of depression, anxiety, and stress, the Depression Anxiety Stress Scale–42 (DASS), which was adapted in Turkish by Bilgel and Bayram [10], was used and Cronbach’s α values were calculated to assess the internal consistency of the scale; they were 0.92, 0.86, and 0.88 for depression, anxiety, and stress, respectively.

Height, weight, and waist circumference were measured by an experienced dietitian. Body mass index (BMI) (kg/m^2^) was calculated using the measured weight and height. Body composition analysis was carried out using professional Tanita BC-418MA hand-to-foot bioelectrical impedance analysis (BIA). Body composition measurement was carried out by providing the required conditions by allowing individuals to stand in the same position for 30 s, in an upright and forward-facing position on the device, without speaking or moving. The individual’s total body water (kg), muscle mass (kg), bone mineral mass (kg), and body fat (%) were determined by measuring from hand to foot with the Tanita BC-418MA. 

### 2.3. Statistical Analysis

The data obtained from this study were analyzed using the SPSS (Statistical Package for Social Sciences) 20.0 package program. Results are expressed as frequencies (n) and percentages (%) for qualitative variables. Quantitative variables are shown as mean ± standard deviation ($\bar{X}\;\pm$ SD). The Shapiro–Wilk test was used to check the normality of distributions. Either a t-test or Mann–Whitney U test was used to analyze the continuous variables. Pearson chi-square (X^2^) analysis and Fisher’s exact test were used in the analysis of categorical variables. In the evaluation of statistical analyses, *p* < 0.05 level was accepted as statistically significant. 

## 3. Results

Participansts’ mean age was 56.6 ± 8.25 years. Approximately half had a higher education level and the prevalence of obesity and overweight were 12.9% and 71%, respectively (Table 1).

Table 2 shows menopausal symptoms and the total scores on the PSQI and DASS according to time elapsed since menopause. Hot flashes, night sweats, and mood swing were more common in women who have been through menopause for less than 5 years. Also, the PSQI, Depression, and Stress scores of those women were higher (* *p* < 0.05). 

Table 3 shows the distribution of sleep conditions of participants as recorded by the PSQI. Women who had been menopausal for 5 or more years had better subjective sleep quality and lower daytime dysfunction according to subgroups of the PSQI (* *p* < 0.05).

Anthropometric measurements of participants according to menopause period are summarized in Table 4. The women who had been menopausal for 5 or more years had a higher BMI, waist/hip ratio, and fat mass (* *p* < 0.05). 

**Figure 1:**
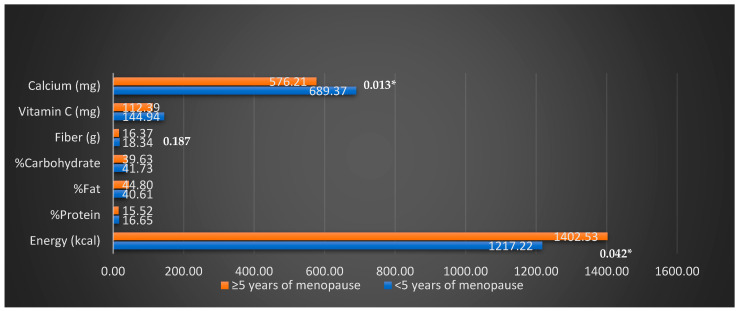
Energy, macro-, and micronutrients intakes of participants according to menopause period.

Energy and fat intake were higher, while protein, vitamin C, and calcium intakes were lower in women who had been menopausal for 5 or more years (* *p* < 0.05) (Figure 1).

## 4. Discussion

Cardiometabolic, physical, and psychosocial health changes can negatively affect the overall life quality of postmenopausal women [11]. These important health changes require dietary and lifestyle interventions employed by healthcare professionals [12]. This study aimed to rule out the healing effect of antidepressant use by focusing specifically on menopausal women using antidepressants and to investigate the factors that can be regulated, such as nutrition and sleep, encountered in early (<5 years) and late (≥5 years) menopause periods.

As shown in Table 2, menopausal symptoms tend to continue despite antidepressant treatment and still present in the majority (about half) of women after 5 or more years. While all menopause-related symptoms decreased, sleep disturbances, fatigue, and weight gain were found to be similar (*p* > 0.05). Valiensi et al. [13] reported a decrease in hot flashes after a 5-year period; however, this difference was not statistically significant, similar to this study, and there was no difference in night sweats. Despite the use of antidepressants, the frequency of severe depression and stress according to DASS in the first 5 years of menopause decreased in late menopause (*p* < 0.05). This result supports the high incidence of mental distress in the early stages of menopause [14,15].

Considering the long-term cardiometabolic risks, ensuring appropriate weight and sleep quality in these women is important for health [16]. The women who had been menopausal for 5 or more years had better subjective sleep quality and lower daytime dysfunction according to subgroups of the PSQI (*p* < 0.05). However, the PSQI sleep score remaining high in late menopausal women (>5 years) is evidence that regulation is needed. Postmenopausal women demonstrate a loss of circadian robustness and sleep. Indeed, menopause alters the gene expression in adipose tissue, possibly related to the redistribution of adiposity. Disrupted circadian rhythm is associated with obesity and cardiovascular and metabolic diseases [17]. According to a study conducted in Korea with 1002 women, 618 of whom were premenopausal and 384 of whom were postmenopausal, the risk of metabolic syndrome increased up to 14 years since menopause, then decreased. For its individual components, postmenopausal women with 5 to 9 years since menopause had the highest risk of high blood pressure; postmenopausal women with less than 5 years since menopause had an increased risk of abdominal obesity and high glucose. With 10 to 14 years since menopause, postmenopausal women had an increased risk of high triglycerides [18]. 

Sleep problems were observed in the majority of menopausal women. In particular, the presence of vasomotor symptoms and eating habits affect sleep status. Moreover, these symptoms can be significantly improved by reducing dietary risk factors. The relationship between a high-fat diet and poor sleep quality is well known [19]. According to the results of this study, it is noteworthy that the nutritional status of women in the late stages of menopause deteriorated rather than improved. Energy and fat intake were higher, while protein, vitamin C, and calcium intakes were lower in women who had been menopausal for 5 or more years.

Since 83.9% of the study group was overweight or obese, sleep disorders and fatigue symptoms tended to continue in more than half of the group, regardless of the time of menopause. The difference in parameters such as BMI, waist circumference, waist/hip ratio, and fat mass was significant between the early and late periods of menopause. Huang et al. reported that losing only 5 kg of weight improves the tolerability of hot flashes by 30% [20]. The risk of Type 2 DM can increase 2.5 times in individuals with evening chronotypes compared with morning types, regardless of sleep quality and duration [21]. Nutritional quality plays a role in maintaining the robustness of the circadian system. There may be a disruption in circadian rhythm in people with eating disorders [22]. 

Better sleep quality in late menopausal women and the selection of patients already using antidepressants draw attention to the importance of nutrition in terms of persistence of symptoms and increase in obesity. Three-day food records are one of the most recommended methods for determining nutritional status; we think that this is the strength of this study. The limitation of this study is mainly its cross-sectional design, which fails to provide any explanation of the causality association. It is not possible to generalize the results of this study, which does not have a very high sample size. However, including individuals who have been diagnosed with psychiatric diseases during menopause and determining their sleep quality and nutritional status by valid methods adds precious value to the study. Furthermore, this was a single-center study which may limit the transfer of these data to other populations. Studies with larger samples are needed on this subject. 

## 5. Conclusions

It is crucial for healthcare professionals to review their approaches to early and late menopausal periods and to individualize all treatment options in patients whose symptoms persist. Dietitians should take part in the process with a multidisciplinary approach in order to improve the quality of life of menopausal women. Eating habits should be regulated and a healthy lifestyle should be encouraged, thus contributing to the reduction of menopausal symptoms, sleep disorders, and depression.

## Figures and Tables

**Table 1:** Sociodemographic and anthropometric data of the participants.

	Women (n = 62)	
	$\bar{X}$	SD
**Age (years)**	56.6	8.25
	**n**	**%**
**Education**		
≤High school	33	53.2
≥College	29	46.8
**Employment status**		
Worker	16	25.8
Non-worker	46	74.2
**Marital status**		
Married	43	69.4
Non-married	19	30.6
**Smoking status**		
Smoker	12	19.4
Non-smoker	50	80.6
**Nutritional Supplements Usage**		
Yes	25	40.3
No	37	59.7
Supplements (n = 35) **		
Vitamin D	28	45.1
Multivitamins	10	16.1
Omega 3	6	9.6
Zinc	3	4.8
Vitamin B_12_	4	6.4
**BMI groups**		
<24.9 kg/m^2^	10	16.1
25.0–29.9 kg/m^2^	44	71.0
≥30.0 kg/m^2^	8	12.9

** Multiple choices.

**Table 2:** Menopausal symptoms, sleep, depression, anxiety, and stress characteristics of participants according to menopause period.

	<5 Years of Menopause n = 29		≥5 Years of Menopause n = 33		*p*
	n	%	n	%	
Menopause Symptoms					
Hot flashes	24	82.8	18	54.5	0.018 *
Night sweats	23	79.3	15	45.5	0.006 *
Weight gain	20	69.0	18	54.5	0.245
Sleeping disorders	18	62.1	18	54.5	0.549
Mood swing	21	72.4	13	39.4	0.009 *
Fatigue	18	62.1	18	54.5	0.549
**PSQI scores**					
Good (<5)	7	24.1	19	57.6	0.008 *
Difficulties (≥5)	22	75.9	14	42.4	
**PSQI (X ± SD)**	6.17 ± 2.20		4.81 ± 2.27		0.021 *
**DASS scores**					
**Depression (X ± SD)**	11.41 ± 7.72		8.09 ± 7.49		0.045 *
Normal	13	44.8	22	66.7	0.060 *
Mild	3	10.3	5	15.2	
Moderate	9	31.0	4	12.1	
Severe	4	13.8	1	3.0	
Extremely Severe	-	-	1	3.0	
**Anxiety (X ± SD)**	6.06 ± 4.50		7.33 ± 4.62		0.294
Normal	20	69.0	21	63.6	0.434
Mild	4	13.8	2	6.1	
Moderate	3	10.3	7	21.2	
Severe	2	6.9	3	9.1	
Extremely Severe	-	-	-	-	
**Stress (X ± SD)**	17.17 ± 9.03		13.09 ± 6.40		0.041 *
Normal	13	44.8	22	66.7	0.017 *
Mild	2	6.9	3	9.1	
Moderate	6	20.7	7	21.2	
Severe	8	27.6	1	3.0	
Extremely Severe	-	-	-	-	

**Table 3:** Sleep conditions of participants as recorded by PSQI.

	<5 Years of Menopause n = 29		≥5 Years of Menopause n = 33		
	S	%	S	%	*p*
**Subjective sleep quality**					
Very good	2	6.9	9	27.3	0.030 *
Fairly good	16	55.2	18	54.5	
Fairly bad	10	34.5	5	15.2	
Very bad	1	3.4	1	3.0	
**Sleep latency**					
<15 min	7	24.1	5	15.2	0.284
16–30 min	10	34.5	14	42.4	
31–60 min	12	41.4	11	33.3	
>60 min	-	-	3	9.1	
**Sleep duration**					
>7 h	12	41.4	17	51.5	0.657
6–7 h	15	51.7	15	45.5	
5–6 h	1	3.4	-	-	
<5 h	1	3.4	1	3.0	
**Sleep efficiency**					
>85%	23	79.3	25	75.8	0.923
75–84%	5	17.2	7	21.2	
65–74%	-	3.4	1	3.0	
<65%	1	-	-	-	
**Sleep disturbance**					
Not during past month	1	3.4	1	3.0	0.666
Less than mina week	20	69.0	26	78.8	
Once or twice a week	8	27.6	6	18.2	
Three or more times a week	-	-	-	-	
**Use of sleep medication**					
Not during hmonth	28	96.6	32	32	0.365
Less than once a week	1	3.4	-	-	
Once or twice a week	-	-	-	-	
Three or more times a week	-	-	**-**	**-**	
**Daytime dysfunction**					
Not during past month	19	65.5	12	36.4	0.044 *
Less than once a week	7	24.2	16	48.5	
Once or twice a week	3	10.3	2	6.1	
Three or more times a week	-	-	3	9.1	
Not during past month					

**Table 4:** Anthropometric measurements of participants according to menopause period.

	<5 Years of Menopause n = 29		≥5 Years of Menopause n = 33		*p*
	$\bar{X}$	SD	$\bar{X}$	SD	
**Anthropometrics**					
BMI (kg/m^2^)	26.66	4.15	28.89	4.84	0.044 *
Waist circumference (cm)	83.89	9.40	92.39	14.23	0.023 *
Hip circumference (cm)	99.51	5.67	104.15	13.42	0.445
Waist/hip ratio	0.85	0.05	0.87	0.03	0.041 *
Total body water (kg)	32.77	3.65	34.00	3.76	0.247
Fat-free mass (kg)	8.81	0.90	8.82	1.28	0.277
Fat mass (%)	33.13	5.13	36.26	4.02	0.026 *

## Data Availability

According to the results, we can share Excel and SPSS files containing the data with Google Drive.
